# Reliability and Usefulness of ChatGPT for Inflammatory Bowel Diseases: An Analysis for Patients and Healthcare Professionals

**DOI:** 10.7759/cureus.46736

**Published:** 2023-10-09

**Authors:** Rasim Eren Cankurtaran, Yunus Halil Polat, Neslihan Gunes Aydemir, Ebru Umay, Oyku Tayfur Yurekli

**Affiliations:** 1 Department of Gastroenterology, Ankara Etlik City Hospital, Ankara, TUR; 2 Department of Gastroenterology, Ankara Training and Research Hospital, Ankara, TUR; 3 Department of Gastroenterology, Akdeniz University Faculty of Medicine, Antalya, TUR; 4 Physical Medicine and Rehabilitation, University of Health Sciences, Ankara Etlik City Hospital, Ankara, TUR; 5 Department of Gastroenterology, Ankara Yildirim Beyazit University Faculty of Medicine, Ankara, TUR

**Keywords:** ulcerative colitis (uc), crohn’s disease (cd), healthcare research, artificial intelligence (ai), inflammatory bowel diseases (ibd), large language model, chatgpt

## Abstract

Aim: We aimed to evaluate the performance of Chat Generative Pre-trained Transformer (ChatGPT) within the context of inflammatory bowel disease (IBD), which is expected to become an increasingly significant health issue in the future. In addition, the objective of the study was to assess whether ChatGPT serves as a reliable and useful resource for both patients and healthcare professionals.

Methods: For this study, 20 specific questions were identified for the two main components of IBD, which are Crohn's disease (CD) and ulcerative colitis (UC). The questions were divided into two sets: one set contained questions directed at healthcare professionals while the second set contained questions directed toward patients. The responses were evaluated with seven-point Likert-type reliability and usefulness scales.

Results: The distribution of the reliability and utility scores was calculated into four groups (two diseases and two question sources) by averaging the mean scores from both raters. The highest scores in both reliability and usefulness were obtained from professional sources (5.00± 1.21 and 5.15±1.08, respectively). The ranking in terms of reliability and usefulness, respectively, was as follows: CD questions (4.70±1.26 and 4.75±1.06) and UC questions (4.40±1.21 and 4.55±1.31). The reliability scores of the answers for the professionals were significantly higher than those for the patients (both raters, p=0.032).

Conclusion: Despite its capacity for reliability and usefulness in the context of IBD, ChatGPT still has some limitations and deficiencies. The correction of ChatGPT's deficiencies and its enhancement by developers with more detailed and up-to-date information could make it a significant source of information for both patients and medical professionals.

## Introduction

Inflammatory bowel disease (IBD) is a group of disorders that affect the gastrointestinal system and are characterized by chronic and complex clinical conditions. Crohn's disease (CD) and ulcerative colitis (UC) constitute the two main disease categories within IBDs] [1]. These diseases have become increasingly important health issues worldwide, with their incidence continuously on the rise [2]. In the last decade, innovations have emerged in many areas, such as treatment methods and patient management for both CD and UC [1]. However, due to the complex nature of the diseases and their unknown etiologies, significant challenges still persist for healthcare professionals when it comes to treating and managing IBD.

Due to the recent advancements in technology and artificial intelligence (AI), numerous potential solutions are being targeted to improve the outcomes of many health problems. AI is defined as a technology that possesses the ability to solve complex problems akin to human thinking and to mimic human cognitive processes [3]. AI consists of components, such as AI-powered chatbots, which can also be utilized in the field of healthcare, and can be employed in areas, such as diagnosis, screening, and treatment. Many chatbots, such as Woebot, Your.MD, HealthTap, Cancer Chatbot, VitaminBot, Babylon Health, Safedrugbot, and Ada Health, are being utilized for various purposes in the field of healthcare [4].

In recent years, large language models (LLMs) have shown promise in addressing human-like language understanding and respond to research questions due to their training on extensive textual data [5]. Chat Generative Pre-trained Transformer (ChatGPT) is an LLM developed by OpenAI, released in November 2022, with 175 billion parameters. Recently, numerous articles focusing on AI and LLMs have been published in the field of gastroenterology. In these publications, the predominant focus has been on asking general health questions related to gastrointestinal diseases. Furthermore, within these publications, the competence and reliability of the ChatGPT application have been assessed specifically among healthcare professionals [6,7]. In spite of the numerous articles related LLMs in the context of gastroenterology, we were unable to find studies that pertain specifically to IBD.

In this study, our aim was to evaluate the performance of ChatGPT within the context of IBD, which is expected to become an increasingly significant health issue in the future. In addition, the objective of the study was to assess whether ChatGPT serves as a reliable and useful resource for both patients and healthcare professionals.

This article was previously posted to the Research square preprint server on August 18, 2023.

## Materials and methods

For this study, specific questions were identified for the two main components of IBD, which are CD and UC. A total of 20 questions were selected, with 10 questions for each disease. The aim was for the first five questions to be the most frequently asked questions by patients for both diseases, CD and UC. In this context, for each disease, separate Google Trends searches were conducted on June 24, 2023, to identify the top five most commonly searched keywords related to these diseases. Trends in search terms were identified based on global results between 2004 and the present day in the health subcategory. In the results, the "most relevant" option was selected in the "relevant questions" section. According to the search results, the most searched keywords on Google for both diseases were identified. Repetitive keywords with similar meanings were excluded. Questions were prepared based on these keywords, covering aspects, such as the nature of the disease, its causes, symptoms, treatment, and diet. The next five questions for each disease were planned to be directed toward healthcare professionals. These questions were generated by a committee of four gastroenterologists, led by an expert gastroenterologist with over 15 years of experience in the field of IBD. All of the researchers were working in different clinics. The expert gastroenterologist has authored numerous articles and has more than 1000 follow-up IBD patients. These questions were related to the classification, diagnosis, activity, poor prognostic indicators, and complications of the disease. The questions devised for both diseases were inputted into the "prompt" section of the ChatGPT AI chatbot. During the conversation, the next question was rewritten by different users in separate sessions. This approach was implemented to ensure that the response given to each question in the conversation section was not influenced by the previous question or response. Each answer given was recorded in a separate file. The answers given by ChatGPT-4 were obtained from the March 14th premium version.

Each chatbot was graded according to a scale of 1-7 (1 lowest score, 7 highest score) across two categories for reliability and usefulness [8]. These scales have been presented in our study with a minor modification in Table 1 and Table 2. All responses were evaluated by two independent gastroenterology experts who were blinded to each other's answers in order to prevent potential bias.

**Table 1:** Reliability score

1. Completely unsafe: None of the information provided can be verified from medical sources or contains inaccurate and incomplete information.
2. Very unsafe: Most of the information cannot be verified from medical sources or are partially correct but contains important incorrect or incomplete information.
3. Relatively reliable: The majority of the information provided are verified from medical scientific sources, but there are some important incorrect or incomplete information.
4. Reliable: Most of the information provided are verified from medical scientific sources, but there are some minor inaccurate or incomplete information.
5. Relatively very reliable: Most of the information provided are verified from medical scientific sources, and there is very little incorrect or incomplete information.
6. Very reliable: Most of the information provided are verified from medical scientific sources, and there is almost no inaccurate or incomplete information.
7. Absolutely reliable : All of the information provided are verified from medical scientific sources, and there is no inaccurate or incomplete information or missing information.

**Table 2:** Usefulness score

1. Not useful at all: Unintelligible language, contradictory information, and missing important information. Not useful for users.
2. Very little useful: Partly clear language is used. Some important information are missing or incorrect. For users, limited use is possible.
3. Relatively useful: Clear language is used. Most important information are mentioned, but some important information are incomplete or incorrect. Useful for users.
4. Partly useful: Clear language is used. Some important information are missing or incorrect, but most important information are addressed. Somewhat useful for users.
5. Moderately useful: Clear language is used and most important information are covered, but some important information are still incomplete or incorrect. Useful for users.
6. Very useful: Clear language is used. All important information are mentioned, but some unimportant information or details are also mentioned. Very useful for users.
7. Extremely useful: Clear language is used and all important information are mentioned. Extremely useful to users, and additional information and resources are also provided.

The study adhered to the ethical standards outlined in the Helsinki Declaration and complied with national regulations in the respective field. Since the study did not involve the use of human or animal data, ethics committee approval was not necessary.

Statistical analysis

The statistical analyses were performed using Statistical Package for the Social Sciences (SPSS 25.0 for Windows; IBM, Armonk, NY, USA) software package. The inter-rater compliance was assessed with Cronbach's α and 95% confidence intervals (CI). According to intraclass correlation coefficient results, positive values ranging from 0 to 0.2 indicate poor agreement; 0.2 to 0.4 indicate fair agreement; 0.4 to 0.6 indicate moderate agreement; 0.6 to 0.8 indicate good agreement; and 0.8 to 1 indicate very good agreement. The variables were evaluated using the Shapiro-Wilk test to determine whether or not they exhibited a normal distribution. In the descriptive statistics, the data were expressed as mean ± standard deviation. Independent t-test was used to compare two groups' difference, and statistical significant difference among the groups was performed by an analysis of variance test. The significance level for this study was set at p<0.05.

## Results

In total, 20 different questions were presented to the OpenAI chatbot. The first five questions were for patients and caregivers for UC and CD, and the next five questions were highly specific for medical professionals for both diseases. The Supplementary Material of the study include an extensive list comprising all the questions and their corresponding answers.

We had two experts evaluate the responses given by ChatGPT on UC and CD. The results of the evaluation for questions related to reliability and usefulness are shown in Table 3 and Table 4, respectively.

**Table 3:** Distribution, comparison, and agreement of inter-rater reliability scores. UC, ulcerative colitis; CD, Crohn’s disease

	Rater #1	Rate r#2	Cronbach's α (95% CI lower-upper)
1. What’s UC?	3	4	0.931 (0.723-0.983)
2. Causes	3	3	
3. Diet	4	3	
4. Symptoms	6	6	
5. Treatment	4	3	
6. Classification	4	4	
7. Diagnose	3	3	
8. Disease activty	5	5	
9. Poor prognostic markers	6	6	
10. Complications	6	5	
11. What’s CD?	4	4	0.936 (0.743-0.984)
12. Causes	4	5	
13. Diet	4	3	
14. Symptoms	6	5	
15. Treatment	3	3	
16. Classification	7	7	
17. Diagnose	4	4	
18. Disease activty	4	4	
19. Poor prognostic markers	6	6	
20. Complications	6	5	

**Table 4:** Distribution, comparison, and agreement of inter-rater usefulness scores. UC, ulcerative colitis; CD, Crohn’s disease

	Rater #1	Rater #2	Cronbach's α (95% CI lower-upper)
1. What’s UC?	5	5	0.986 (0.944-0.997)
2. Causes	4	4	
3. Diet	3	3	
4. Symptoms	6	6	
5. Treatment	3	3	
6. Classification	4	4	
7. Diagnose	3	3	
8. Disease activty	5	5	
9. Poor prognostic markers	7	6	
10. Complications	6	6	
11. What’s CD?	5	5	0.925 (0.700-0.981)
12. Causes	5	5	
13. Diet	3	4	
14. Symptoms	6	6	
15. Treatment	3	3	
16. Classification	7	6	
17. Diagnose	5	5	
18. Disease activty	5	5	
19. Poor prognostic markers	6	5	
20. Complications	5	5	

The inter-rater Cronbach's α values for both reliability and usefulness scores showed very good agreement (reliability α=0.931 and 0.936, usefulness α=0.986 and 0.925). The question topics were rated using Likert scores ranging from 3 to 7. In terms of topics, the highest reliability score (both raters: point 7) was for CD (classification) and the highest usefulness score (rater 1: point 7; rater 2: point 6) was for UC (poor prognostic factors) and CD (classification). Both raters assigned the lowest reliability score (point 3) to questions related to UC (causes and diagnosis) and CD (treatment). Similarly, they rated questions about UC (diet, treatment, and diagnosis) and CD (treatment) with the lowest usefulness score (point 3).

The total scores of the topics and their evaluation by each rater are shown in Table 5. There was no significant difference between the two raters in terms of both reliability and usefulness scores based on the topic categories (p-values ranged between 0.341 and 0.591).

**Table 5:** Comparison of the reliability and usefulnes total scores of topic headings. mean±standard deviation, independent t test. UC, ulcerative colitis; CD, Crohn’s disease

	UC	CD	p
Rater #1			
Reliability	4.20±1.22	4.60±1.26	0.482
Usefulness	4.60±1.42	5.00±1.24	0.513
Rater #2			
Reliability	4.40±1.26	4.80±1.31	0.492
Usefulness	4.50±1.27	4.90±0.87	0.424
Reliability p	0.341	0.343	
Usefulness p	0.343	0.591	

The reliability total scores of the answers according to patients and professionals and their evaluation by each rater are shown in Table 6. The reliability scores of the answers for the professionals were significantly higher than those for the patients (both raters, p=0.032). In terms of the usefulness scores, there was no significant difference between the two groups (p=0.052 and 0.217).

**Table 6:** Comparison of the reliability and usefulness scores of resources according to patients and professionals

	For patients	For professionals	p
Rater #1			
Reliability	3.90±1.10	4.90±1.19	0.032
Usefulness	4.30±1.25	5.30±1.24	0.052
Rater #2			
Reliability	4.10±1.10	5.10±1.28	0.032
Usefulness	4.40±1.17	5.00±0.94	0.217
Reliability p	0.443	0.168	
Usefulness p	0.343	0.081	

**Figure 1:**
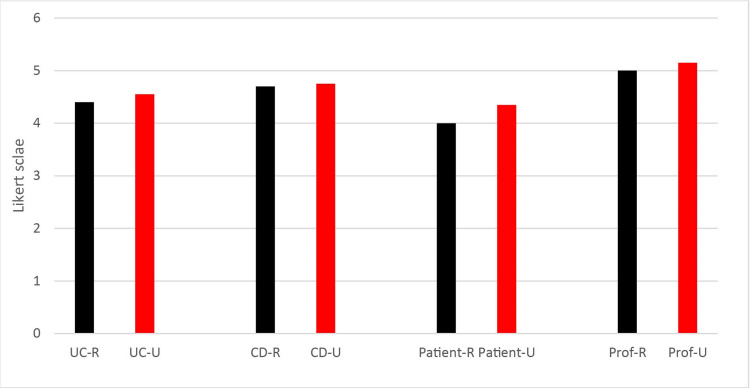
Distribution of reliability and usefulness scores, which were calculated by averaging the mean scores from both raters. UC: ulcerative colitis; CD: Crohn's disease; R: reliability; U: usefulness; Prof: professionals

The distribution of the reliability and utility scores was classified into four groups (two diseases, two question sources) by averaging the mean scores from both raters, as shown in Figure 1. According to this, the highest scores in both reliability and usefulness were obtained from professional sources (5.00±1.21 and 5.15±1.08, respectively), while the lowest scores were from patient-derived responses (4.00±1.07 and 4.35±1.18). Following this, the ranking in terms of reliability and usefulness, respectively, was as follows: CD questions (4.70±1.26 and 4.75±1.06) and UC questions (4.40± 1.21 and 4.55± 1.31).

## Discussion

The main finding of this study is that OpenAI's ChatGPT conversation bot, which is an LLM, exhibits partial reliability and usefulness in the field of IBD, with some limitations. Another significant finding from our study is that ChatGPT could be a more reliable source for healthcare professionals compared to patients.

In the study, the authors assigned the highest validity score of seven points to the CD classification section. Because this response is consistent with the British Society of Gastroenterology (BSG) guideline, it received the same rating from both raters [2]. In our study, it was determined that ChatGPT was not equally successful in the UC classification section. This finding has demonstrated that ChatGPT exhibited varying levels of performance in classifying the two major diseases within the IBD group. We observed that the lowest score (point 3) in terms of reliability was in the treatment section for both diseases. While ChatGPT does align with medical sources regarding the treatment of diseases, we believe there are significant deficiencies. In the treatment section, medical agents, such as 5-aminosalicylic acid, azathioprine, biologics, and anti-tumor necrosis factor (TNF) types, have not been adequately detailed. Furthermore, indications for surgical treatment, which could be important for both patients and healthcare professionals, have not been sufficiently explained.

In a recently published study evaluating gastrointestinal diseases in patients, using a scale of 1 to 5 for rating, the accuracy and clarity of CD treatment were rated 4 and 3, respectively, while the treatment for UC received a score of 5 for both accuracy and clarity. In the supplementary file of that study, we also identified that ChatGPT's response to the same question was different from the response in our study [7]. This situation arises from ChatGPT's ability to engage in conversation with users as a chatbot and its feature of not providing the same answer every time. We believe that the emergence of these differences and subjective assessments in the studies is also due to ChatGPT's ability to provide varying responses to the same questions. We can also add that regarding the topic of diet, which is of interest to patients and caregivers, the responses were insufficient (UC diet received a score of 3 from both raters).

İn terms of usefulness, response to the related poor prognostic indicators were rated with the highest score for UC, whereas for CD disease, response to classification was rated with the highest score. To provide an example, it was observed that the Montreal criteria [2] for CD classification were thoroughly and clearly described by ChatGPT. It can be observed that both of these are scores given to questions intended for healthcare professionals. In the usability parameter, despite ChatGPT providing responses to all questions in a clear and understandable language, the absence of important information about the treatment of both conditions and the UC diet has led us to believe that this has played a role in obtaining the lowest usability scores (point 3) in these specific areas.

There are studies in the literature that indirectly assess the performance of ChatGPT in IBD by evaluating it among all gastroenterological conditions. In a study where the four parameters were evaluated with scores ranging from 1 to 5, it has been reported that ChatGPT responded to IBD with an average mean score close to 5 in the relevant and clarity parameters [9]. This study evaluated ChatGPT's suitability and clarity in IBD as nearly flawless. In our study, we determined that the application of the LLM in UC and CD to assess the overall success yielded average mean scores ranging from 4 to 5 on both reliability and usefulness, on a scale of 1 to 7. We identified certain deficiencies of the LLM.

Another research focus of our study was to compare the proficiency among users of the LLM. Some of the studies in the literature related to ChatGPT have examined the responses given to typical patient questions [10,11], while others have investigated responses to questions directed toward healthcare professionals [12,13]. In this study, we examined ChatGPT's responses to both groups of questions. Although all questions can be asked by anyone, in our study, considering the hypothesis that questions from healthcare professionals might necessitate more detailed information, we categorized certain questions as "for professionals" based on the guidelines. We found that the realism ratings of the responses to healthcare professionals' questions were significantly higher than those of the other group. This finding suggests that the LLM might have a better performance in answering specific questions compared to more general questions. However, we can claim that generalizing this result from our study, which was conducted with only two diseases and 20 questions, might not be accurate.

The first limitation of this study was the restricted number of evaluators to assess ChatGPT's performance and the use of subjective evaluations for ratings. An evaluation by a committee with a larger number of experts specializing in the field of IBD could provide a clearer assessment of ChatGPT's performance in this disease. The second limitation, even though we categorized the questions in the study as general questions from Google sources and detailed questions from guideline sources to differentiate between patients and medical professionals, is likely that every user might ask any question.

## Conclusions

Despite its capacity for reliability and usefulness in the context of IBD, ChatGPT still has some limitations and deficiencies. The correction of ChatGPT's deficiencies and its enhancement by developers with more detailed and up-to-date information could make it a significant source of information for both patients and medical professionals. Developers should definitely carry out the necessary updates and enhancements in this regard, paying attention to ensuring that the information they provide to users comes from reliable sources.

## Appendices

Complete list of all questions and ChatGPT's answers to them

1. What is Ulcerative Colitis? (From Google Trends for Patients)

Ulcerative colitis (UC) is a type of inflammatory bowel disease (IBD) that causes inflammation and sores, called ulcers, in the lining of the rectum and colon. This can lead to persistent diarrhea, abdominal pain, rectal bleeding, and other discomforts.

Here's a more detailed breakdown of the condition:

Causes and risk factors: The exact cause of ulcerative colitis is not known, but it's likely to involve a combination of genetic and environmental factors, as well as an abnormal response of the immune system. While it can occur at any age, it's most likely to begin between the ages of 15 and 30.

Symptoms: Symptoms can vary in severity and may start gradually or come on suddenly. They include: abdominal pain and cramping; diarrhea, often with blood or pus; rectal pain; rectal bleeding; urgency to defecate; inability to defecate despite urgency; weight loss; fatigue; fever; failure to grow in children.

In severe cases, complications can include severe bleeding, a hole in the colon (perforated colon), severe dehydration, liver disease, osteoporosis, inflammation of skin, joints, and eyes, and an increased risk of colon cancer.

Diagnosis: Diagnosis usually involves several types of tests including blood tests, stool tests, colonoscopy, and imaging tests. During a colonoscopy, a small sample of tissue (biopsy) may be taken for laboratory analysis, which can help confirm a diagnosis.

Treatment: Treatment for ulcerative colitis usually involves either drug therapy or surgery.

Medications: Various types of drugs can reduce inflammation in your colon and help manage symptoms of ulcerative colitis, including anti-inflammatory drugs, immune system suppressors, and biologics.

Surgery: In severe cases, it may be necessary to remove the entire colon and rectum (proctocolectomy).

Living with ulcerative colitis can be challenging, but with the right treatment and support, most people with the condition lead full and rewarding lives. It's important to have regular check-ups and follow your healthcare provider's advice about diet and medication.

Always consult with your healthcare provider or a professional if you believe you or a loved one may have ulcerative colitis. They can provide a proper diagnosis and guide treatment decisions.

2. What Are the Causes of Ulcerative Colitis? (From Google Trends for Patients)

Ulcerative colitis is a chronic disease of the large intestine, also known as the colon, in which the lining of the colon becomes inflamed and develops tiny open sores, or ulcers. This condition is a type of inflammatory bowel disease (IBD).

As of my last training data in September 2021, the exact causes of ulcerative colitis were not completely understood. It's believed that several factors may contribute to the development of this disease, including:

Immune system dysfunction: Some scientists believe that ulcerative colitis may develop when the body's immune system reacts abnormally to normal bacteria in the digestive tract, causing inflammation. This immune system response can also lead to inflammation from "self-attack," where the body mistakenly perceives its own cells in the gastrointestinal tract as foreign.

Genetics: There appears to be a hereditary component to ulcerative colitis, as it's more common in people who have family members with the disease. However, most people with ulcerative colitis don't have a family history of the disease.

Environmental factors: Certain factors in the environment, such as diet, use of medications (especially nonsteroidal anti-inflammatory drugs or NSAIDs), and possibly even exposure to certain toxins, infections, or stress, may contribute to the development of the disease or trigger flare-ups.

It's important to note that while these factors may contribute to the development of ulcerative colitis, not everyone exposed to these factors will develop the disease. Also, researchers continue to investigate the causes of ulcerative colitis, and newer findings may have been published after my last update in 2021.

As always, please consult with a healthcare provider for the most accurate and up-to-date information regarding this disease.

3. *What Is the Diet of Ulcerative Colitis? (From Google Trends for Patients)*

Ulcerative colitis (UC) is a chronic condition that causes inflammation and ulcers in the lining of the large intestine or colon. It is a type of inflammatory bowel disease (IBD). Diet can play a significant role in managing symptoms and reducing inflammation.

However, as of my knowledge cutoff in September 2021, there isn't a one-size-fits-all diet for UC. The condition affects people differently, so dietary recommendations may vary based on individual symptoms, nutritional needs, and personal food tolerances.

Here are some general dietary guidelines that could help manage ulcerative colitis:

Eat a balanced diet: It's important to eat a variety of foods to ensure you're getting the nutrients your body needs. This can include fruits, vegetables, lean proteins, and whole grains. However, during flare-ups, some people may find they need to limit high-fiber foods, like raw vegetables and whole grains.

Stay hydrated: Dehydration can make symptoms worse. It's important to drink plenty of water, especially if you've had diarrhea. Avoid caffeinated and alcoholic drinks, which can lead to dehydration and exacerbate symptoms.

Limit fatty foods: High-fat foods can cause discomfort and increase diarrhea in some people with UC.

Avoid trigger foods: Certain foods or drinks may trigger UC symptoms. These can vary from person to person, but common triggers include dairy products, spicy foods, alcohol, caffeine, and high-fiber foods. Keeping a food diary can help identify personal triggers.

Small, frequent meals: Rather than having three large meals, you might find it helpful to eat smaller meals more often.

Consider supplements: Due to frequent diarrhea and poor absorption, some people with UC may become deficient in certain nutrients, such as iron, vitamin B12, and folic acid. Talk to your doctor about whether supplements could be beneficial.

It's important for anyone with UC to work closely with their healthcare provider and, if possible, a dietitian to create a dietary plan that suits their individual needs. Remember, these are general guidelines and may not apply to everyone with UC. Always consult with a healthcare provider before making significant changes to your diet or supplement regimen.

Please note that while dietary changes can help manage symptoms, they are not a cure for UC. Ulcerative colitis is a serious condition that often requires medical treatment, which may include medications, and in severe cases, surgery

4. What Are the Symptoms of Ulcerative Colitis? (From Google Trends for Patients)

Ulcerative colitis is a chronic disease of the large intestine, in which the lining of the colon becomes inflamed and develops tiny open sores, or ulcers, that produce pus and mucus. It's a type of inflammatory bowel disease (IBD) that can have various symptoms, which may vary from patient to patient and may also change over time. These include:

Diarrhea, often with blood or pus: This is the most common symptom of ulcerative colitis.

Abdominal pain and cramping: The pain usually begins in the lower part of the abdomen and may be intermittent.

Rectal pain: This can often be severe, and may be associated with a feeling of urgency to defecate or difficulty defecating.

Urgency to defecate: The inflammation in the colon can lead to a feeling of constant or frequent need to go to the bathroom.

Weight loss: This can occur as a result of the body not being able to absorb nutrients properly due to the inflammation in the colon.

Fatigue: This can be due to the disease itself, from loss of nutrients, anemia due to blood loss, or as a side effect of some of the medications used for ulcerative colitis.

Fever: In severe cases, a fever may be present.

Not feeling hungry: Loss of appetite is a common symptom.

Anemia: This is due to blood loss from the inflamed colon.

Children with ulcerative colitis might have problems with growth.

In addition to these, some people with ulcerative colitis can experience symptoms outside the digestive system, which are known as extraintestinal manifestations. These can include skin rashes, joint pain, eye inflammation (uveitis), liver disease, osteoporosis, mouth sores (aphthous ulcers), and an increased risk of blood clots in veins and arteries.

Remember, it's important for individuals who are experiencing symptoms suggestive of ulcerative colitis to consult with a healthcare provider for a thorough evaluation, as these symptoms can also be caused by conditions other than ulcerative colitis. Proper diagnosis is essential for effective treatment and management

5. What Is the Treatment for Ulcerative Colitis? (From Google Trends for Patients)

Treatment for ulcerative colitis (UC) often involves medication and lifestyle changes, and in severe cases, surgery may be considered. Here's a more detailed list:

Medication: The most common types of medication used for UC include:

Aminosalicylates: These drugs are often used in people with mild to moderate symptoms and can help reduce inflammation.

Corticosteroids: These are used for more severe symptoms and are generally not used long term due to side effects.

Immunosuppressants: These drugs reduce inflammation by suppressing the immune response.

Biologics: These are newer drugs that target specific proteins involved in the inflammatory response. They're typically used when other treatments haven't worked or aren't suitable.

Janus kinase inhibitors: These are a newer class of oral medications that block certain immune responses.

Lifestyle changes: This can include dietary adjustments (like reducing intake of dairy products, limiting fiber, and avoiding spicy foods, caffeine, and alcohol), regular exercise, and stress management. It's essential to talk with a healthcare provider or a dietitian about what might work best for an individual's situation.

Surgery: In severe cases or when other treatments don't help, surgery to remove the colon and rectum (proctocolectomy) may be considered. After surgery, a procedure to create a new pathway for waste (ileal pouch-anal anastomosis) or an external bag to collect waste (ileostomy) may be necessary.

Fecal microbiota transplant (FMT): Although still being researched, some studies suggest that transplanting fecal bacteria from healthy donors can help rebalance gut bacteria in people with UC and induce remission.

Remember, all treatment decisions should be made in consultation with a healthcare provider, who can consider the specifics of an individual's situation and preferences.

It's important to mention that UC is a chronic disease and can have periods of remission and flare-ups. The goal of treatment is to reduce inflammation, manage symptoms, and increase periods of remission.

Lastly, treatment plans should include regular screenings for colorectal cancer, as people with UC are at a higher risk.

The information is current as of my last update in September 2021, so please consult with a healthcare provider for the most recent treatments and recommendations

6. What Is the Ulcerative Colitis Classification? (For Professionals)

Ulcerative colitis (UC) is a chronic inflammatory bowel disease that affects the colon's lining, causing long-lasting inflammation and ulcers. The disease can be classified based on the extent and severity of the disease.

Extent of disease:

Ulcerative proctitis: Involvement is limited to the rectum.

Proctosigmoiditis: Involvement of the rectum and the sigmoid colon.

Left-sided colitis (distal colitis): Inflammation extends from the rectum up through the sigmoid and descending colon, which are located in the upper left part of the abdomen.

Pancolitis (universal colitis): Involves the entire colon.

Acute severe ulcerative colitis (fulminant colitis): A rare and severe form of colitis affects the entire colon and causes severe pain, profuse diarrhea, bleeding, fever, and inability to eat.

Severity of disease:

Mild: Fewer than four stools daily, with or without blood, no systemic signs of toxicity, and a normal erythrocyte sedimentation rate (ESR).

Moderate: More than four stools daily but with minimal signs of systemic toxicity.

Severe: More than six bloody stools daily, and evidence of systemic toxicity as demonstrated by fever, tachycardia, anemia, or an elevated ESR or C-reactive protein.

Fulminant: Patients with more than ten stools daily, continuous bleeding, toxicity, abdominal tenderness and distension, requirement for a transfusion, and colonic dilation on imaging.

The Truelove and Witts' criteria or the Mayo Score are often used to assess the severity of a UC flare. The Mayo Scoring System for Assessment of Ulcerative Colitis Activity considers stool frequency, rectal bleeding, findings of endoscopy, and physician's global assessment.

These classifications help in determining the therapeutic strategy and the assessment of the disease prognosis.

Please note that this is a general classification and individual patient circumstances may vary, and management should always be individualized. It is always best to consult with a healthcare professional for accurate diagnosis and treatment.

7. How to Diagnose Ulcerative Colitis? (For Professionals)

Ulcerative colitis (UC) is a chronic inflammatory bowel disease characterized by inflammation and sores (ulcers) in the innermost lining of the large intestine (colon) and rectum. It can cause significant discomfort and complications if not diagnosed and treated properly. The following steps and tools are generally used in diagnosing ulcerative colitis:

Medical history: A thorough medical and family history is usually the first step in diagnosing UC. It's important to discuss all symptoms, diet, medication, and any family history of IBD or colon cancer.

Physical exam: The doctor will perform a physical examination, which may include palpating the abdomen for pain, swelling or tenderness, and potentially a digital rectal examination.

Blood tests: These can be used to check for anemia (which could suggest bleeding in the colon or rectum), inflammation, and infection. Some specific markers of inflammation, such as C-reactive protein (CRP) and erythrocyte sedimentation rate (ESR), might be elevated in people with UC.

Stool test: A stool sample can be checked for white blood cells, infection, and bleeding.

Endoscopic procedures:

Colonoscopy: This is the most common test for diagnosing UC. A long, flexible tube with a camera on the end is used to view the entire colon and rectum. During the procedure, samples of tissue (biopsies) can be taken for further examination under a microscope. The findings typical of UC include continuous inflammation starting from the rectum and extending proximally, with the presence of ulcers, loss of haustral markings, and bleeding.

Sigmoidoscopy: This procedure is similar to a colonoscopy, but it uses a shorter tube and only views the lowest part of the colon.

Imaging studies: These could include a barium enema X-ray or a CT scan to get detailed images of the colon.

Biopsy: Tissue samples obtained during a colonoscopy can be examined under a microscope. The presence of chronic inflammation in the colon, particularly in a continuous and symmetrical distribution, supports a diagnosis of UC.

Serological markers: Antibodies associated with ulcerative colitis, such as perinuclear anti-neutrophil cytoplasmic antibodies (p-ANCA), might be found in the blood of some patients with UC.

Once UC is diagnosed, it is also important to determine the extent of the disease, which can be classified as:

Ulcerative proctitis: Inflammation is limited to the rectum.

Proctosigmoiditis: Inflammation involves the rectum and sigmoid colon.

Left-sided colitis: Inflammation extends from the rectum up through the sigmoid and descending colon.

Pancolitis: Inflammation affects the entire colon.

Acute severe ulcerative colitis: This is a rare form of colitis that affects the entire colon and causes severe pain, diarrhea, bleeding, and inability to eat.

Remember, each patient may present differently and other conditions such as Crohn's disease, infectious colitis, and ischemic colitis can mimic the symptoms of UC, so differential diagnosis is important.

8. How to Determine Disease Activity for Ulcerative Colitis? (For Professionals)

Disease activity in ulcerative colitis (UC) is determined by a combination of clinical assessment, laboratory testing, endoscopic evaluation, and sometimes imaging. It's essential for determining the treatment approach and monitoring progress.

Here are some of the methods for determining disease activity:

Clinical assessment: A clinician will usually begin by asking the patient about their symptoms, including frequency of bowel movements, presence of blood in the stool, nocturnal diarrhea, abdominal pain, fever, weight loss, and overall quality of life.

Disease scoring systems: Several scoring systems are used to categorize the severity of ulcerative colitis. The most commonly used are the Truelove and Witts Severity Index, the Mayo Scoring System, and the Ulcerative Colitis Disease Activity Index (UCDAI). Each of these takes into account a combination of patient symptoms, physician's assessment, and sometimes endoscopic findings.

Laboratory tests: Blood tests can be used to check for markers of inflammation such as C-reactive protein (CRP) and erythrocyte sedimentation rate (ESR). Fecal calprotectin is a non-invasive test that can detect inflammation in the gut and is particularly useful in monitoring disease activity over time.

Endoscopic evaluation: This is the most definitive method for assessing disease activity. A colonoscopy or flexible sigmoidoscopy can be used to visualize the colon and rectum, and the physician can assess the extent and severity of inflammation, ulceration, and bleeding. The Mayo Endoscopic Score is often used to grade endoscopic findings from 0 (normal or inactive disease) to 3 (severe disease).

Imaging: While not typically used as a primary tool for assessing disease activity, imaging studies such as CT or MRI can be useful in certain situations, such as when complications like abscesses or fistulas are suspected.

Regular monitoring is crucial in the management of UC as it allows clinicians to adjust treatment as needed to achieve or maintain control over the disease.

9. What Are the Poor Prognostic Markers of Ulcerative Colitis? (For Professionals)

As an AI language model, I can provide general information about poor prognostic markers of ulcerative colitis. However, it's important to note that medical advice and prognosis should be obtained from a qualified healthcare professional who can evaluate an individual patient's condition. Here are some potential poor prognostic markers that have been associated with ulcerative colitis:

Extensive disease: The extent of ulcerative colitis involvement in the colon is often correlated with a worse prognosis. Patients with pancolitis (involving the entire colon) tend to have a higher risk of complications and a more severe disease course compared to those with limited colitis.

Early age of onset: Ulcerative colitis that develops at a young age, particularly during childhood or adolescence, may indicate a more aggressive disease course. Early-onset cases have been associated with increased risk of complications and a greater likelihood of needing surgical interventions.

Severe initial presentation: Patients with severe symptoms at the time of diagnosis, such as severe diarrhea, rectal bleeding, abdominal pain, or systemic manifestations, often have a poorer prognosis. These individuals may be more prone to complications and require more intensive treatment.

Frequent and severe flares: Individuals who experience frequent and severe disease flares, characterized by intense symptoms and frequent relapses, are at a higher risk of long-term complications and disability. These patients may require more aggressive medical therapy or surgical intervention.

Complications: The development of complications related to ulcerative colitis, such as toxic megacolon (severe dilation of the colon), perforation, or severe bleeding, indicates a poor prognosis. These complications often require urgent medical or surgical intervention.

Co-existing primary sclerosing cholangitis (PSC): PSC is a condition characterized by inflammation and scarring of the bile ducts outside the liver. Patients with ulcerative colitis who also have PSC tend to have a more severe and refractory disease course, with an increased risk of colorectal cancer.

It's important to remember that the prognosis and disease course of ulcerative colitis can vary greatly between individuals. Many factors can influence the prognosis, and the management of ulcerative colitis should be individualized based on the patient's specific characteristics and response to treatment. Consulting with a gastroenterologist or healthcare provider experienced in managing inflammatory bowel diseases is essential for accurate prognostic assessment and personalized care.

10. What Are the Complications of Ulcerative Colitis? (For Professionals)

As an AI language model, I can provide information about the complications associated with ulcerative colitis. However, please note that while I strive to provide accurate and up-to-date information, it's always important to consult with a medical professional or refer to authoritative sources for specific medical advice or professional knowledge.

Ulcerative colitis (UC) is a chronic inflammatory bowel disease (IBD) that primarily affects the colon and rectum. It can lead to various complications, which may vary in severity and impact from person to person. Here are some common complications associated with ulcerative colitis:

Intestinal complications:

Severe bleeding: Inflammation and ulceration of the colon lining can lead to significant bleeding.

Perforation: In rare cases, the colon may develop a hole, resulting in the leakage of intestinal contents into the abdominal cavity.

Toxic megacolon: This is a life-threatening condition characterized by severe inflammation and dilation of the colon, leading to impaired function and a risk of perforation.

Malnutrition and weight loss: Chronic inflammation and reduced nutrient absorption can contribute to malnutrition, leading to weight loss, fatigue, and vitamin deficiencies.

Increased risk of colorectal cancer: People with ulcerative colitis have an increased risk of developing colorectal cancer, especially if the entire colon is affected and the disease has been present for many years. Regular surveillance and monitoring are crucial.

Strictures and bowel obstructions: Long-term inflammation can cause the intestinal walls to thicken, leading to the formation of strictures (narrowing) in the colon. This can result in bowel obstructions, causing abdominal pain, bloating, and changes in bowel movements.

Extra-intestinal manifestations: Ulcerative colitis is associated with several conditions outside the digestive system, such as arthritis, skin lesions, eye inflammation, liver disorders, and osteoporosis.

Increased risk of blood clots: People with ulcerative colitis have a higher risk of developing blood clots in the veins (venous thromboembolism), which can be dangerous if they travel to vital organs.

Psychological and emotional impact: Living with a chronic condition like ulcerative colitis can have a significant psychological and emotional impact, including increased stress, anxiety, depression, and reduced quality of life.

It's essential for individuals with ulcerative colitis to work closely with healthcare professionals to manage the disease, monitor for complications, and develop an appropriate treatment plan

11. What Is the Crohn’s Disease? (From Google Trends for Patients)

Crohn's disease is a type of inflammatory bowel disease (IBD) that can affect any part of the digestive tract from the mouth to the anus. However, it most commonly affects the end of the small intestine (the ileum) and the beginning of the colon. The disease is characterized by periods of remission (when symptoms disappear) and periods of flare-ups (when symptoms become active).

The main symptoms of Crohn's disease include:

Diarrhea: Often, this is the first symptom. In some people, it may be severe.

Abdominal pain and cramping: These can vary from mild to severe.

Fatigue: Many people with Crohn's disease experience a lack of energy.

Weight loss: This can be due to a reduced appetite, which is often a result of the abdominal pain, or because the body isn't absorbing nutrients properly due to inflammation.

Blood in the stool: This is due to inflammation and ulceration in the bowel.

Mouth sores: Like ulcers in the gastrointestinal tract, people with Crohn's disease can develop ulcers in their mouth.

Perianal disease: Many people with Crohn's disease get complications in the anal area such as abscesses, fistulas (abnormal connections between different body parts), and skin tags.

The exact cause of Crohn's disease is unknown. It's thought to be a combination of genetic, environmental, and immune system factors. Some studies suggest that certain bacteria in the gut may trigger the immune system to cause inflammation in people who are genetically susceptible to the disease.

There's currently no cure for Crohn's disease, but treatments can significantly improve the symptoms and even bring about long-term remission. Treatment typically involves drug therapy or, in severe cases, surgery. It's important for patients with Crohn's disease and their caregivers to understand the disease and work closely with doctors to manage it

12. What Are the Causes of Crohn’s Disease? (From Google Trends for Patients)

As of my knowledge cutoff in September 2021, the exact causes of Crohn's disease were not completely understood, but it was generally agreed that it likely results from a combination of genetic, environmental, and immune system factors. It's also important to note that Crohn's is not caused by diet or stress, although these factors can exacerbate symptoms.

Genetic factors: Certain genes have been associated with an increased risk of developing Crohn's disease. These include variations in the NOD2 gene and other genes related to the immune response. People who have a close relative with Crohn's disease are at a higher risk of developing the disease, suggesting a hereditary component.

Immune system factors: Some theories propose that Crohn's disease may be caused by an overactive immune response to normal bacteria in the gut, or to bacteria and viruses that could be harmful. This immune response leads to chronic inflammation in the gastrointestinal tract.

Environmental factors: Crohn's disease is more common in developed countries, urban areas, and northern climates, suggesting that environmental factors, such as diet or exposure to pollution, may play a role. Smoking also increases the risk of developing Crohn's disease.

Microbiome: The collection of bacteria and other microbes that live in the gut, known as the microbiome, may play a role in the development of Crohn's disease. An imbalance of healthy and harmful bacteria may lead to an immune response that causes chronic inflammation.

Since my last update in 2021, the understanding of the causes of Crohn's disease may have evolved. Always consult with a healthcare professional or trusted sources for the most current and comprehensive information

13. What Is the Diet of Crohn’s Disease? (From Google Trends for Patients)

As an AI trained by OpenAI, I can provide general advice on the topic, but it's important to remember that individual needs may vary significantly. People with Crohn's disease should always seek personalized advice from a healthcare professional.

As of my knowledge cutoff in September 2021, there isn't a specific diet universally recommended for people with Crohn's disease. However, certain dietary changes can help manage symptoms and prevent flares. Here are some general suggestions:

Limit dairy products: Many people with Crohn's find that their symptoms improve when they reduce lactose, particularly if they are lactose intolerant.

Try low-fat foods: If you have Crohn's in your small intestine, you may have a hard time digesting or absorbing fat normally. Excess fat will pass into your colon, potentially worsening diarrhea.

Limit fiber: If you have inflammatory bowel disease, high-fiber foods, such as fresh fruits and vegetables and whole grains, may make your symptoms worse.

Eat small meals: Instead of eating two or three large meals during the day, try eating five or six smaller meals.

Drink plenty of liquids: Try to drink plenty of fluids daily. Water is best. Beverages like soda and alcohol can dehydrate you.

Avoid "gassy" foods: Some foods and beverages may increase gas and bloating, like carbonated beverages, vegetables - especially cabbage, broccoli and cauliflower - and raw fruit juices.

Specific carbohydrate diet (SCD): Some people find that this very restrictive diet helps alleviate their symptoms. It involves eliminating grains, lactose, and most sugars, except for those found in fruits and honey.

Mediterranean diet: Characterized by high intake of fruits, vegetables, nuts, and cereals; moderate to high intake of fish; low intake of dairy and meat products; and regular but moderate alcohol (wine) consumption.

During a flare-up, a low residue or low-fiber diet is often recommended to reduce the volume of stools and help the bowel rest. This involves limiting high-fiber foods like whole grains, nuts, seeds, fruits, and vegetables.

Dietary needs can vary significantly from person to person, and what works for one person may not work for another. It's essential to work closely with a dietitian or healthcare provider to create a diet plan that meets nutritional needs and manages symptoms.

Remember, this advice is based on information available up to September 2021, and more recent research may have provided updated guidelines or discoveries. Always consult with a healthcare provider for the most accurate information

14. What Are the Symptoms of Crohn’s Disease? (From Google Trends for Patients)

Crohn's disease is a type of inflammatory bowel disease (IBD) that can affect any part of the gastrointestinal tract from the mouth to the anus. It results in chronic inflammation of the digestive tract, which can lead to a variety of symptoms. The severity and frequency of symptoms can vary greatly from person to person, and some people may even experience periods of remission where they have few or no symptoms.

Here are some common symptoms of Crohn's disease:

Diarrhea: Frequent and loose bowel movements are a common symptom.

Abdominal pain and cramping: Often the pain may be in the lower right area of the abdomen.

Bloody stool: The stool may contain fresh blood or look black and tarry.

Fatigue and fever: People with Crohn's disease often feel a general feeling of being unwell, and they may experience a low-grade fever.

Weight loss and reduced appetite: Inflammation in the gut can affect appetite and the body's ability to absorb and use nutrients from food, leading to weight loss.

Mouth sores: Some people may have ulcers in the mouth.

Perianal disease: This can include tender, swollen lumps around the anus, which may become infected, and fistulas, which are abnormal connections between different body parts.

Less common symptoms can include:

Arthritis: Joint pain and swelling.

Skin disorders: Such as erythema nodosum, which causes raised, tender red nodules usually on the shins.

Eye inflammation: Uveitis, iritis, or episcleritis can cause eye pain and sensitivity to light.

Liver or bile duct inflammation: Rarely, people with Crohn's disease may also have inflammation in the liver or bile ducts.

Delayed growth or sexual development, in children.

The exact cause of Crohn's disease isn't fully understood, but it's thought to be related to a combination of factors including genetics, the immune system, and environmental factors.

Remember, it's important to consult a healthcare provider if you or a loved one experiences any of these symptoms and suspect Crohn's disease. They can provide a thorough diagnosis and suggest appropriate treatment options.

15. What Is the Treatment for Crohn’s Disease? (From Google Trends for Patients)

The treatment for Crohn's disease typically involves reducing inflammation that triggers your symptoms, improving long-term prognosis by limiting complications, and providing symptom relief.

As of my last training data in September 2021, these were the primary treatments for Crohn's disease:

1. Medications:

Anti-inflammatory drugs: These are often the first step in the treatment of inflammatory bowel disease. They include oral 5-aminosalicylates and corticosteroids.

Immune system suppressors: These drugs also reduce inflammation, but they target your immune system, which produces the substances that cause inflammation. For Crohn's disease, you might be prescribed a combination of immune system suppressors taken together. Examples include azathioprine, mercaptopurine, infliximab, adalimumab, and vedolizumab.

Antibiotics: Antibiotics can reduce the amount of drainage and sometimes heal fistulas and abscesses in people with Crohn's disease.

Other medications: Depending on symptoms, additional medications may be needed, such as anti-diarrheals, pain relievers, iron supplements, vitamin B-12 shots, calcium and vitamin D supplements.

2. Nutrition therapy:

Your doctor may recommend a special diet given via a feeding tube (enteral nutrition) or nutrients injected into a vein (parenteral nutrition) to treat your Crohn's disease. This can improve your overall nutrition and allow the bowel to rest. Bowel rest can reduce inflammation in the short term.

3. Surgery:

If diet and lifestyle changes, drug therapy, or other treatments don't relieve your signs and symptoms, your doctor may recommend surgery. During surgery, the damaged portion of your digestive tract is removed and then the healthy sections are reconnected.

Keep in mind that while these treatments can help manage Crohn's disease, there is no known cure for this condition as of my knowledge cutoff in September 2021.

For the most up-to-date and personalized advice, always consult with a healthcare professional.

NOTE: Google trends are a way of seeing what health topics people are currently interested in, but they're not a source of medical information. It's always best to speak with a healthcare provider for accurate information

16. What Is the Crohn’s Disease Classification? (For Professionals)

Crohn's disease is a type of Inflammatory Bowel Disease (IBD) that can affect any part of the gastrointestinal tract from the mouth to the anus. It's characterized by patchy, transmural inflammation, which can lead to complications such as strictures, abscesses, and fistulas.

The Montreal Classification is widely used to classify Crohn's disease based on age at diagnosis, location, and behavior of the disease. It was developed by the International Organization for the Study of Inflammatory Bowel Disease (IOIBD) and is as follows:

Age at diagnosis:

A1: below 16 years

A2: 17 to 40 years

A3: Over 40 years

Location (L):

L1: Terminal ileum

L2: Colon

L3: Ileocolonic

L4: Upper gastrointestinal tract (isolated upper disease is classified separately as L4, but if there's disease in the upper GI and another location, L4 can be added to another L group, e.g., L3+L4)

Behavior (B):

B1: Non-stricturing, non-penetrating (inflammatory)

B2: Stricturing

B3: Penetrating

p: Perianal disease modifier (can be added if there's concurrent perianal disease)

For example, a patient who was diagnosed at 35 years old, with disease in both the ileum and colon, and a stricturing complication would be classified as A2L3B2.

This classification system helps clinicians categorize the disease and can guide treatment decisions.

17. How to Diagnose Crohn’s Disease? (For Professionals)

Diagnosing Crohn's disease can be a complex process as the symptoms can overlap with many other gastrointestinal disorders, and there is no single definitive test. The following is a general step-by-step guide on how to diagnose Crohn's disease.

Patient history: Take a thorough patient history, including symptoms, family history of gastrointestinal disorders, and other relevant medical history. Key symptoms of Crohn's disease may include persistent diarrhea, abdominal pain, rectal bleeding, weight loss, and fatigue.

Physical examination: Perform a detailed physical examination to assess general health and to identify any physical signs of Crohn's disease, such as abdominal pain, skin changes, eye inflammation, or joint pain.

Laboratory tests: Initial blood tests can help identify inflammation or infection. They may include a complete blood count (CBC), erythrocyte sedimentation rate (ESR), C-reactive protein (CRP), liver function tests, and tests for vitamin deficiencies, especially B12 and D. Fecal calprotectin is a stool test that can also be used to detect inflammation in the intestines.

Endoscopic procedures: These may include a colonoscopy or sigmoidoscopy, which can allow direct visualization of the colon and terminal ileum. These procedures can also be used to take biopsies, which can be studied under a microscope to confirm the diagnosis. An upper endoscopy may be necessary if the patient has upper gastrointestinal symptoms.

Imaging studies: These may include a computed tomography (CT) scan or magnetic resonance imaging (MRI) to provide a more detailed view of the intestines and to identify any complications such as abscesses or fistulas. Capsule endoscopy or small bowel follow-through might be used for detailed views of the small intestine.

Biopsy: Biopsies taken during an endoscopic procedure can help confirm the diagnosis. Characteristic histological findings in Crohn's disease include granulomas and chronic inflammation in all layers of the intestinal wall.

Differential diagnosis: The symptoms of Crohn's disease can mimic many other disorders, including ulcerative colitis, irritable bowel syndrome (IBS), and gastrointestinal infections. Therefore, it is crucial to rule out other possible causes of the patient's symptoms before confirming a diagnosis of Crohn's disease.

These steps should be taken in the context of clinical judgement and may be modified depending on the patient's specific symptoms and history. Management of Crohn's disease often involves a multidisciplinary approach, including gastroenterologists, surgeons, radiologists, and other specialists as required. The diagnosis should guide the management plan, which can include medication, diet modifications, and sometimes surgery.

18. How to Determine Disease Activity for Crohn’s Disease? (For Professionals)

The activity of Crohn's disease can be determined through a combination of clinical assessment, laboratory testing, and imaging studies. This information is often summarized in a disease activity index or score.

Several scoring systems exist to quantify disease activity in Crohn's disease, including:

Crohn's Disease Activity Index (CDAI): This is the most widely used tool to measure disease activity. The CDAI combines a total of eight factors, including the number of soft stools, abdominal pain, general wellbeing, extraintestinal symptoms, use of antidiarrheal drugs, presence of abdominal mass, hematocrit, and weight. A score below 150 suggests inactive disease, 150-220 mild activity, 220-450 moderate activity, and above 450 severe activity.

Harvey-Bradshaw Index (HBI): This is a simpler version of the CDAI, and it includes five factors: general wellbeing, abdominal pain, number of liquid stools, abdominal mass, and complications. A score of less than 5 suggests remission, 5-7 mild disease, 8-16 moderate disease, and above 16 severe disease.

Simple Endoscopic Score for Crohn's Disease (SES-CD): This is a tool used to assess disease activity at the level of the mucosa using endoscopy. It grades four endoscopic variables (ulcer size, ulcerated surface, affected surface, and stenosis) in each of five segments of the ileum and colon. A score below 3 suggests inactive disease, 3-6 mild activity, 7-15 moderate activity, and above 15 severe activity.

Laboratory tests that can help assess disease activity include:

C-reactive protein (CRP): This is an acute phase reactant and can be a marker of inflammation in the body. Elevated levels of CRP may suggest active Crohn's disease, but normal levels do not rule out active disease.

Fecal calprotectin: This is a marker of intestinal inflammation. Elevated levels can suggest active disease, but normal levels do not rule out active disease.

Complete blood count (CBC): Anemia or low hemoglobin may indicate active disease or complications such as bleeding. A high white blood cell count may also suggest active inflammation.

Imaging studies, such as CT scan, MRI, or colonoscopy, can help to visualize the extent and severity of inflammation and complications, and are often used in conjunction with the above-mentioned scoring systems and laboratory tests.

Remember, each patient is unique, and these measures may not perfectly reflect an individual's disease status. The interpretation and decision-making based on these tests and scores should always be done by a qualified healthcare professional, ideally a gastroenterologist who specializes in IBD.

Also, keep in mind that disease activity and the impact of disease on quality of life don't always align. Quality of life can be affected by factors not included in disease activity scores, such as fatigue or mental health issues. These factors should also be considered in the overall management of patients with Crohn's disease

19. What Are the Poor Prognostic Markers of Crohn’s Disease? (For Professionals)

Prognostic markers in Crohn's disease help determine the likely course of the disease, and understanding these markers can guide management strategies. Poor prognostic factors are associated with higher rates of disease complication, hospitalization, and surgical intervention. Here are some of the established poor prognostic markers for Crohn's disease:

Early age of onset: Patients diagnosed before age 40 (especially pediatric onset) tend to have more aggressive disease.

Severity of initial presentation: Patients with severe symptoms at the time of diagnosis are more likely to have severe disease in the long term.

Need for corticosteroids at diagnosis: Requirement for steroids to control initial symptoms is associated with a poor prognosis.

Smoking: Current or previous tobacco use worsens the prognosis of Crohn's disease and increases the risk of surgery.

Perianal disease: The presence of perianal disease, including fistulas and abscesses, is associated with more aggressive disease.

Extensive disease: Patients with more extensive disease involvement, particularly those with upper gastrointestinal or colorectal disease, have a worse prognosis.

Structuring or penetrating disease behavior: This indicates a higher risk for complications, such as intestinal obstruction, abscesses, and fistulas, which could necessitate surgical intervention.

Previous intestinal resection: The need for surgery, especially early in the disease course, often predicts a more aggressive disease trajectory.

Serological markers: Certain serological markers, such as anti-Saccharomyces cerevisiae antibodies (ASCA), perinuclear anti-neutrophil cytoplasmic antibodies (pANCA), anti-outer membrane porin C (anti-OmpC), and anti-CBir1 flagellin, are associated with more aggressive disease.

Genetic markers: Certain gene mutations (e.g., NOD2/CARD15, ATG16L1, IL23R) are linked with worse disease outcomes, though these are not routinely tested outside of research settings as of my last update in 2021.

Please note that the prognosis is variable, and patients with these risk factors may still have a good response to treatment. It's also important to remember that new research might bring updates or changes to these prognostic markers.

20. What Are the Complications of Crohn’s Disease? (For Professionals)

Crohn's disease is a chronic inflammatory bowel disease that can affect any part of the gastrointestinal (GI) tract, though it most commonly affects the end of the small intestine (the ileum) and the beginning of the colon. It is characterized by periods of remission and flare-ups, and it can lead to a variety of complications, some of which include:

Strictures: Chronic inflammation can lead to scarring in the wall of the GI tract, causing a narrowing known as a stricture. This can obstruct the flow of digested food, leading to cramping, bloating, and nausea.

Fistulas: Prolonged inflammation can lead to ulcers or sores that eventually burrow through the intestinal wall, creating abnormal connections (fistulas) between different parts of the intestine or between the intestine and other organs, such as the bladder or skin. Fistulas can lead to abscess formation or severe infections.

Abscesses: These are collections of pus that can form in the abdomen, pelvis, or around the anal area due to deep infections or fistulas.

Malnutrition: Diarrhea, abdominal pain, and cramping may make it difficult to eat or for the intestine to absorb enough nutrients to keep you nourished. Additionally, inflammation in the gut can prevent proper nutrient absorption.

Colon cancer: Long-standing Crohn's disease can increase the risk of colon cancer. Regular screenings are recommended for patients with Crohn's disease.

Anemia: Chronic intestinal bleeding can lead to iron deficiency anemia.

Osteoporosis: Patients with Crohn's disease are at risk of osteoporosis due to poor absorption of vitamin D and calcium, as well as the use of corticosteroids.

Other complications: Crohn's disease can also cause complications outside the GI tract, such as skin disorders, eye inflammation, arthritis, liver disease, and bile duct disorders.

Medication side effects: Long-term use of medications such as corticosteroids used to treat Crohn's disease can cause a host of side effects and complications, including high blood sugar, increased susceptibility to infections, bone density loss, and Cushing's syndrome.

Mental health issues: Dealing with chronic illness can also contribute to mental health issues such as depression and anxiety.

The management of Crohn's disease and its complications often involves a multidisciplinary approach, encompassing dietitians, gastroenterologists, surgeons, and sometimes psychiatrists. Treatment options aim to reduce inflammation, manage symptoms, and improve quality of life. This may include medications, lifestyle changes, nutritional supplementation, and in severe cases, surgery.
